# What Is the Best Method for Preventing Infection of Operative Wounds?

**Published:** 1890-12

**Authors:** 


WHAT IS THE BEST METHOD FOR PREVENTING INFECTION OF OPERATIVE WOUNDS?—In a paper read before the Illinois State Medical Society, Dr. L. M. McArthur stated that one-half of the primary wounds, under the present methods, are dressed aseptically at the time of operation, and then only become infected at the redressing. Operators under excitement are inclined to drop into careless habits, and proceed somewhat after the following fashion: They call for a questionable basin, and dropping into it an indefinite amount of carbolic acid proceed to remove the dressings without any such formalities as we were satisfied were essential at first. Here is where the fallacy lies to-day. Too great carelessness at the redressing permits infection and encourages the skeptical in the belief that there is nothing in the principles of aseptic surgery. Before the old dressing is removed a stream of 1 to 1,000 should be ready and playing on the inner layer of gauze as it is being removed, and during the time of exposure of the wound. Having rendered the parts clean, they can best be kept so by providing, in addition to the regular dressing, a heavy dressing of absorbent cotton, not with the idea of catching discharge, but with the object of filtering the atmosphere which is to gain access to the wound through the dressings.—*Medical Record.*

---

Gottschalk, of Berlin, recommends the following formula or vomiting of pregnancy:

| | | | |
|---|---|---|---|
| ℞. | Menthol, | - - - - | grains xvj. |
| | Sps. Vini, | - - - - | drachms vj. |
| | Aq. Dest., | - - - - | ounces v. |

Sig. One tablespoonful every hour.

—*Ex.*

---

A spray of chloroform, 10, ether, 15, menthol, 1, produces complete anæsthesia of the skin lasting for from two to six minutes.—*Exchange.*

